# Mandibular asymmetry: literature review and case report

**DOI:** 10.1590/S1808-86942012000400028

**Published:** 2015-10-20

**Authors:** Belmiro Cavalcanti do Egito Vasconcelos, Fabio Gonçalves, Aureo Andrade, Milagros Guillen, Fabricio Landim

**Affiliations:** 1 PhD (Graduate Program Coordinator at UPE); 2 MD (Medical Resident in Surgery and Maxillofacial Trauma. Universidade de Pernambuco); Universidade de Pernambuco

**Keywords:** maxillofacial abnormalities, mouth abnormalities, tooth abnormalities

## INTRODUCTION

Soft and bone tissue facial asymmetry is seen in patients with and without facial cosmetic alterations. The etiology is believed to be related to congenital, developmental, or acquired factors. In some cases, asymmetry may be secondary to condylar hyperplasia or hypoplasia, anchylosis, or hemifacial microsomia. Seventy-four percent of orthodontically treated patients present chin deviation^1^, ^2^.

The growth of the skull, maxilla, and mandible are closely related. If growth is decompensated in one of these areas, the asymmetric growth and development of part of the craniofacial skeleton may result in a chin deviated from the mandibular midline. Patients with deviated chins usually present asymmetries in other portions of the facial skeleton. Genetic and trauma-related asymmetries may involve muscles, produce excessive unilateral growth, or adversely affect mandible development^3^.

Hemimandibular asymmetry often leads to chin deviation, which by its turn may produce malocclusion and consequently functional and masticatory disorders. Schmid et al.^4^ reported that 28% to 70% of facial asymmetry patients with deviated chins had structural asymmetry, while only 10% had pure asymmetry. Ferrario et al.^5^ have found varying degrees of soft tissue asymmetry in patients without alterations and normal teeth. Facial asymmetry is considered to be present even in normal craniofacial complexes, and a *cant* of 0-3 mm may be deemed normal in healthy unaffected patients^6^. The diagnosis of facial asymmetry is carried out mainly with the aid of cephalometric measurements, clinical examination, cast models and photographs^6^, ^7^.

Prevalence rates of facial asymmetry range between 21% and 85%^1^, ^6^. The variation on the prevalence rates may stem from sample characteristics, dental-facial deformity type, assessment methods and tools, and the criteria defining asymmetry used by the authors. The structures on the lower third of the face are usually more asymmetric than those in the middle third. According to the literature, the left side of the face is usually more affected due to genetic predisposition^8^.

Patients are referred to surgery on an each case basis, depending on the unique features of their involvement, cant extension in the plane of occlusion of the maxilla, tilt angle of the plane of occlusion of the maxilla and mandible, and chin asymmetry. Surgery can be done on the maxilla and mandible, on the mandible alone, on the mandible combined with genioplasty, or genioplasty alone, depending on the patient's case^1^.

## CASE DESCRIPTION

Patient M.G., 22, male, arrived at the Maxillofacial Trauma Surgery Service at XX complaining of a deviated chin.

Physical examination showed he had class III malocclusion and a deviation in the mandibular dental midline in relation to the chin midline. Skull posteroanterior and profile x-ray views revealed the patient had mandibular asymmetry characterized by an elongated mandibular branch.

**Figure 1:**
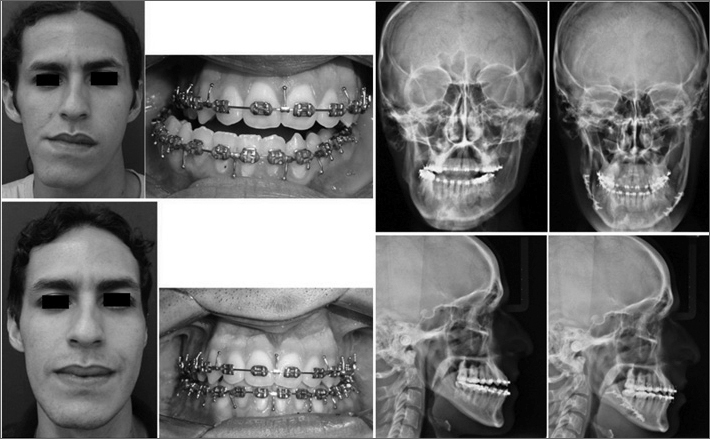
Patient pictures and X-ray images before and after surgery.

The patient was offered a mandibular bilateral sagittal osteotomy, and had 5 mm of bone removed on the left side and 1 mm on the right side. The patient has been followed up for three months and is very pleased with the outcome of the procedure. Patient pictures and x-ray images before and after surgery can be seen in Figure 1.

## DISCUSSION

According to the literature, mandibular asymmetry is more prevalent on the left side. However, the patient described in this case report had deviation on his right side^8^.

Facial asymmetry prevalence rates are high among patients with class III malocclusion. Patients in this situation require repair surgery. Class III molar relationship may be present in one of both sides. Similar studies, as the one done on the Japanese population, indicate that more than 85% of the patients with class III skeletal malocclusion have facial asymmetry and deviated bone structure midlines^8^. All these patients had some sort of directional asymmetry such as vertical elongation of the mandible or maxilla and a deviated chin on the contralateral side^8^. The case reported in this paper matches such findings, as the patient had vertical elongation of the left mandibular branch, chin deviated to the right, and class III malocclusion only on the left side.

Facial asymmetry surgery is challenging, given that it involves handling soft and bone tissue, and often includes the maxilla, mandible, chin, and combinations thereof. Age-based planning is controversial, as condylar growth usually persists until 18 to 23 years of age. Primary care is delivered through a combination of orthodontics and surgery to repair bone defects, while soft tissue is followed up after physiological muscle adaptation^2^.

Mandibular bilateral sagittal osteotomy combined with mentoplasty is the treatment of choice for facial asymmetry in cases where the maxilla is untouched and for patients whose chin midline does not match the mandibular dental midline. In the case reported the mandibular dental midline was deviated to the right by 9 mm from the maxillary midline, and the chin midline was 2 mm off in relation to the mandibular dental midline. However, our patient was offered only a mandibular bilateral sagittal osteotomy, as bone asymmetry was repaired during surgery and no asymmetry was seen between the chin the patient's mandibular midline. Mild chin deviation was observed during immediate postoperative care, and the patient was offered a second procedure to repair it. Nonetheless, the patient was extremely happy with the first procedure and believes no further intervention is required. “I think things are great now. It's perfect. My lower lip feels discretely numb. My right TMJ used to click, but it doesn't anymore. To me, my bite is normal. My friends cannot see it, and I do not need more surgery.”

The surgeon and the orthodontist must agree to repair minimal misalignments (up to 3 mm) and to perform less invasive procedures, i.e., more than one surgical procedure. The risks and benefits related to the procedure must be considered as the patient's care strategy is planned.

## CONCLUSION

Patients with class III malocclusion combined with facial asymmetry in which the mandibular midline does not match the chin midline require mandibular bilateral sagittal osteotomy. However, the patients' perceptions over their condition must be taken into account.
